# The Trim family of genes and the retina: Expression and functional characterization

**DOI:** 10.1371/journal.pone.0202867

**Published:** 2018-09-12

**Authors:** Rebecca Chowdhury, Lauren A. Laboissonniere, Andrea K. Wester, Madison Muller, Jeffrey M. Trimarchi

**Affiliations:** Department of Genetics, Development and Cell Biology, Iowa State University, Ames, Iowa, United States of America; Instituto Murciano de Investigacion y Desarrollo Agrario y Alimentario, SPAIN

## Abstract

To better understand the mechanisms that govern the development of retinal neurons, it is critical to gain additional insight into the specific intrinsic factors that control cell fate decisions and neuronal maturation. In the developing mouse retina, *Atoh7*, a highly conserved transcription factor, is essential for retinal ganglion cell development. Moreover, *Atoh7* expression in the developing retina occurs during a critical time period when progenitor cells are in the process of making cell fate decisions. We performed transcriptome profiling of *Atoh7*+ individual cells isolated from mouse retina. One of the genes that we found significantly correlated with *Atoh7* in our transcriptomic data was the E3 ubiquitin ligase, *Trim9*. The correlation between *Trim9* and *Atoh7* coupled with the expression of *Trim9* in the early mouse retina led us to hypothesize that this gene may play a role in the process of cell fate determination. To address the role of *Trim9* in retinal development, we performed a functional analysis of *Trim9* in the mouse and did not detect any morphological changes in the retina in the absence of *Trim9*. Thus, *Trim9* alone does not appear to be involved in cell fate determination or early ganglion cell development in the mouse retina. We further hypothesize that the reason for this lack of phenotype may be compensation by one of the many additional TRIM family members we find expressed in the developing retina.

## Introduction

The retina is a powerful tool for studying the central nervous system and has been intensively investigated for over a century[1]. It is organized as a laminar tissue, comprised of six different neuronal cell types and one glial cell type. These functionally and morphologically diverse groups of cells arise from a pool of multipotent retinal progenitor cells (RPCs)[2–5]. In the murine retina, neurogenesis begins at about embryonic day (E)11.5. Birthdating studies have demonstrated that the retinal ganglion cells (RGCs) are the first retinal neurons to be born, followed closely by cone photoreceptors, horizontal cells and then amacrine cells[6–9]. The bipolar cells and Müller glia are born later in development, while rod photoreceptors are generated nearly throughout the developmental process[6–9]. One key question that arises in this context is how RPCs that are yet to choose a cell fate make the decision to generate a particular cell type. In an effort to better understand the process of cell fate determination in the retina, single cell transcriptomes of RPCs at various developmental stages were analyzed[10]. Mining these transcriptomes revealed a large number of new marker genes and a significant amount of gene expression heterogeneity, particularly among transcription factors[10].

One such transcription factor was the well-studied Atonal homolog 7 (*Atoh7)*, a basic helix-loop-helix (bHLH) transcription factor whose expression in RPCs is temporally correlated with cell cycle exit and cell fate specification[11]. Furthermore, loss of *Atoh7* in the vertebrate retina leads to an almost complete loss of RGCs[12–16]. However, overexpression experiments have been more equivocal. For example, retinal explants infected with an *Atoh7* expressing retrovirus did not produce more RGCs[17], but other studies testing the effects of *Atoh7* overexpression in Müller glia or stem cells reported increases in RGC generation[18,19]. Finally, lineage tracing studies have shown that other early born retinal neurons besides RGCs also arise from *Atoh7*+ cells. Taken together, these experiments suggest that ATOH7 regulates the competence of RPCs to potentially generate different neurons rather than directly driving the choice to become a particular neuronal type[13,20,21] and other factors are most likely involved in the process of RGC specification.

In an attempt to identify other factors involved in the cell fate specification of early-generated retinal cell types, the transcriptomes of single mouse RPCs were examined. Upon examination of these transcriptomes, we found that one of the most highly represented gene families in these cells was the Tripartite motif (*Trim*) family. In the current study, we have examined the expression of different *Trim* family genes in the developing mouse retina. Through a combination of microarray profiling and *in situ* hybridization (ISH), we found 24 different *Trim* family genes expressed during early retinal development in the mouse. Since *Atoh7* expression is associated with RGC competence [20,21], we decided to focus on genes whose expression was correlated with *Atoh7*, hypothesizing that they may play a role in deciding the fate of retinal neurons during development[22–24]. Of the *Trim* family genes, the expression of *Trim9* was both highly correlated with *Atoh7* by gene clustering and was observed in subsets of *Atoh7*+ single cell transcriptomes. Furthermore, the heterogeneity of *Trim9* expression indicated that its potential role in the retina might affect only a subset of cells.

TRIM9, a member of the tripartite motif containing (TRIM) family of E3 ubiquitin ligases, has been found in the developing and adult central nervous system[25,26]. TRIM9 immunoreactivity was shown to be diminished in affected brain areas in Parkinson’s disease and dementia with Lewy bodies, indicating a possible role for TRIM9 in neurodegenerative diseases[25]. Analysis of a *Trim9* deficient mouse established that TRIM9 mediates the axonal outgrowth of cortical neurons in response to NETRIN-1 through interactions with DCC[26]. Specifically, in the absence of TRIM9, cortical axons showed exaggerated branching and a reduced sensitivity to NETRIN-1[26]. More recently, it was demonstrated that TRIM9 ubiquitinates VASP, an actin regulatory protein located at the tips of filopodia, to produce a spatial gradient of filopodial stability required for the axon turning toward netrin, thereby regulating axon pathfinding in the cortex[27]. In addition to these molecular and cellular phenotypes, severe deficits in spatial learning and memory were observed in *Trim9* knockout mice[28]. In this study, we examined the development of the retina in the absence of *Trim9*. Unlike in the cortex, we observed no significant alteration of retinal morphology upon disruption of *Trim9*. We thoroughly examined the RGCs in these mice and also detected no changes, including in a number of RGC subtypes. Given the substantial number of additional *Trim* family genes expressed in the developing retina, it could be either that *Trim9* is not required for cell fate determination or that compensatory mechanisms exist within this gene family in the developing retina.

## Materials and methods

### Ethics statement

All procedures for the care and housing of mice conform to the U.S. Public Health Service Policy on the Humane Care and Use of Laboratory Animals and were approved by the Institutional Animal Care and Use Committee at Iowa State University.

### Mouse genotyping

The generation of *Trim9* deficient mice has been described previously [26]. Specific primers were used to detect the KO band [F: 5’—CTTCTGAGGGTTGGAGAAAAGC—3’ and R: 5’-CGTGAGAGCTGCTTTCTTATTGG- 3’] and the WT band [F: 5’—CTTCTGAGGGTTGGAGAAAAGC—3’ and R: 5’—CGACGGTATCGATAAAGCTAGCTTGG—3’]. All three primers were used in the same reaction according to the following program: 3 min. at 95°C; then 37 cycles of 1 min. at 94°C, 1 min. at 58°C, 90 seconds at 72°C; followed by 10 min. at 72°C. The WT and KO PCR product sizes were 410 bp and 346 bp, respectively, and were separated on a 2% agarose gel.

### Tissue processing for cryosectioning

Retinas were collected from adult mice as well as mice from 3 different stages of development, ranging from E12.5 to E16.5. Pregnant females were euthanized, embryos collected, and heads incubated in 4% paraformaldehyde (PFA) in phosphate buffered saline (PBS) overnight (O/N) at 4°C. Similarly, adult WT and *Trim9* KO littermate pairs were euthanized, and the eyes placed in 4% PFA/PBS overnight. The eyes were subjected to three 15 min. washes in PBS, after which the retinas were isolated and rocked in 30% sucrose in PBS until they sank. OCT solution (Tissue-Tek) was added at a 1:1 ratio with the 30% sucrose in PBS and rocked until the solution reached equilibration. Retinas were stored at -80°C until they were cryosectioned at 20μm and placed onto Superfrost Plus microscope slides.

### *In situ* hybridization (ISH)

Riboprobe synthesis was performed as described previously[22]. Briefly, RNA probes (650–800 bp in length) were synthesized by PCR amplification using primers specific for mouse cDNA, listed in S1 Table. The probe template sequences were cloned into pGEM-T vector (Promega) and sequenced. Antisense riboprobes were synthesized using either T7 or SP6 RNA Polymerase (depending on clone orientation) in the presence of Digoxigenin for 1–2 hours at 37°C. Riboprobes were treated with DNase I (RNase-free, Roche) for 15 min. and precipitated with 100% Ethanol and LiCl O/N.

*In situ* hybridization on retinal cryosections was performed as described previously [29]. Briefly, the slides were washed with PBS three times, fixed with 4% PFA in PBS, acetylated and the riboprobes hybridized O/N at 65°C. The next day, slides were incubated in a 1X SSC [diluted from 20X saline sodium citrate- 3M NaCl, 0.33M Sodium Citrate, pH 7] buffer containing 50% formamide, treated with RNase A and washed with 2X and 0.2X SSC. After washing twice with TNT (0.1 M Tris-HCl, pH 7.5, 0.15 M NaCl, 0.05% Tween-20), slides were blocked for an hour with 20% heat inactivated sheep serum (HISS) and incubated with anti-DIG-alkaline phosphatase (anti-DIG-AP) antibody (1:2500, Roche) O/N. The next day slides were washed with TNT and developed using NBT and BCIP. Finally, the slides were fixed in 4% PFA/PBS and mounted with Fluoromount-G (Southern Biotech).

### Section immunohistochemistry [IHC]

Slides containing cryosections were incubated for 30 min. with blocking solution (1% BSA, 0.01% Triton X-100, 0.004% SDS) and then placed in primary antibody, diluted according to manufacturer’s instructions in blocking solution. Slides were washed in blocking solution three times for 15 min. at room temperature. Slides were then placed in secondary antibody, diluted 1:300 with blocking solution and incubated at 4°C O/N. Following this incubation, the slides were again washed three times for 15 min. with blocking solution and mounted with Fluoromount-G.

### Whole-mount IHC

The process was carried out exactly as described previously[23]. Briefly, mice were euthanized, and the eyeballs fixed in 4% PFA/PBS O/N. After three 15 min. washes, retinas were dissected and equilibrated in increasing concentrations of sucrose (10%, 20% and 30% w/v sucrose) for 20–30 min. each. After the retinas sank in 30% w/v sucrose solution they were subjected to three freeze-thaw cycles on dry ice. The frozen retinas were stored at -80°C until ready to continue with IHC. To proceed, retinas were washed three times for 30 min. with PBS, and then rocked gently in blocking solution [3% goat serum/1% bovine serum albumin (BSA)/0.1% Triton-X100/0.02% sodium dodecyl sulfate (SDS) in PBS] for 2 hours at room temperature. Retinas were then incubated in primary antibody in blocking solution O/N at 4°C on a rocker. The retinas were then washed with PBS three times for 30 min. each and incubated in secondary antibody at a 1:300 dilution in blocking solution overnight at 4°C on a rocker. Retinas were washed three times with PBS for 30 min. each and then flattened between two coverslips and imaged using the Leica SP5 XMP confocal microscope at Iowa State University.

Primary antibodies used were anti-CalbindinD-28K (CALB) (1:2000; Swant, Switzerland), anti-Calretinin (CALR) (1:1000; Millipore), anti-Visual system homeobox-2 (VSX2) (1:1000;[30]), anti-Transcription factor AP-2α (TCFAP2α) (1:200; Santa Cruz Biotechnology), anti-Rhodopsin (RHO4D2) (1:100;[31]), anti-Choline Acetyltransferase (CHAT) (1:100; Millipore), anti-Protein kinase C-alpha (PKCα) (1:10,000; Sigma-Aldrich), anti-Brain-specific homeobox/POU domain protein-3a (BRN3A) (1:500; Chemicon MAB1585), anti-Opsin-4 (OPN4) (1:1000; Advanced Targeting System). Secondary antibodies used were AlexaFluor 488 AffiniPure donkey anti-rabbit IgG, AlexaFluor 488 AffiniPure goat anti-mouse IgG, AlexaFluor 488 AffiniPure donkey anti-goat IgG (Jackson ImmunoResearch laboratories).

### qPCR

qPCR was performed as described previously [23]. RNA was isolated from the retina using Tri-reagent (Sigma) according to manufacturer’s instructions. 400ng of RNA was used to generate cDNA using random primers and SuperScript III (Life Technologies) according to standard protocols. SybrGreenMasterMix (Thermofisher) was used to perform qPCR in a Bio-Rad MiniOpticon cycler, using the following program: 15 min. at 95°C and 40 cycles of15 sec. at 95°C, 30 sec. at 56°C, and 30 sec. at 72°C. The analysis of the qPCR data was performed exactly as described previously[22]. Each sample was normalized to β-actin to obtain the ΔCt values. The difference in ΔCt values between experimental and control was designated as ΔΔCt. The experiments were repeated three times and the average ΔΔCt value was calculated. The base 2 analogs of the average ΔΔCt value represented the fold change. These results were plotted graphically with error bars shown. Primers used for qPCR are listed in S2 Table.

### Microarrays

Microarray hybridization was performed as described previously[23]. Briefly, RNA was isolated from retinas using Tri-reagent (Sigma) according to standard manufacturer’s protocols. 400ng of total RNA was used to generate aRNA, and 5μg was fragmented using the Ambion MessageAmp™ II aRNA Amplification Kit according to manufacturer’s instructions. Samples were hybridized to Affymetrix GeneChip Mouse Genome 430 2.0 arrays at the Gene-Chip facility at the University of Iowa Genetic Division.

Microarray results were analyzed using the Bioconductor Affy package in R [32]. RMA was employed for background adjustment and normalization[33]. Heatmaps were generated using Genesis software[34]. The ShinyGO v0.41 Gene Ontology Enrichment Analysis tool was used to classify genes according to biological process, molecular function and cellular component[35].

## Results

### Retinal expression of TRIM family genes

**Fig 1:**
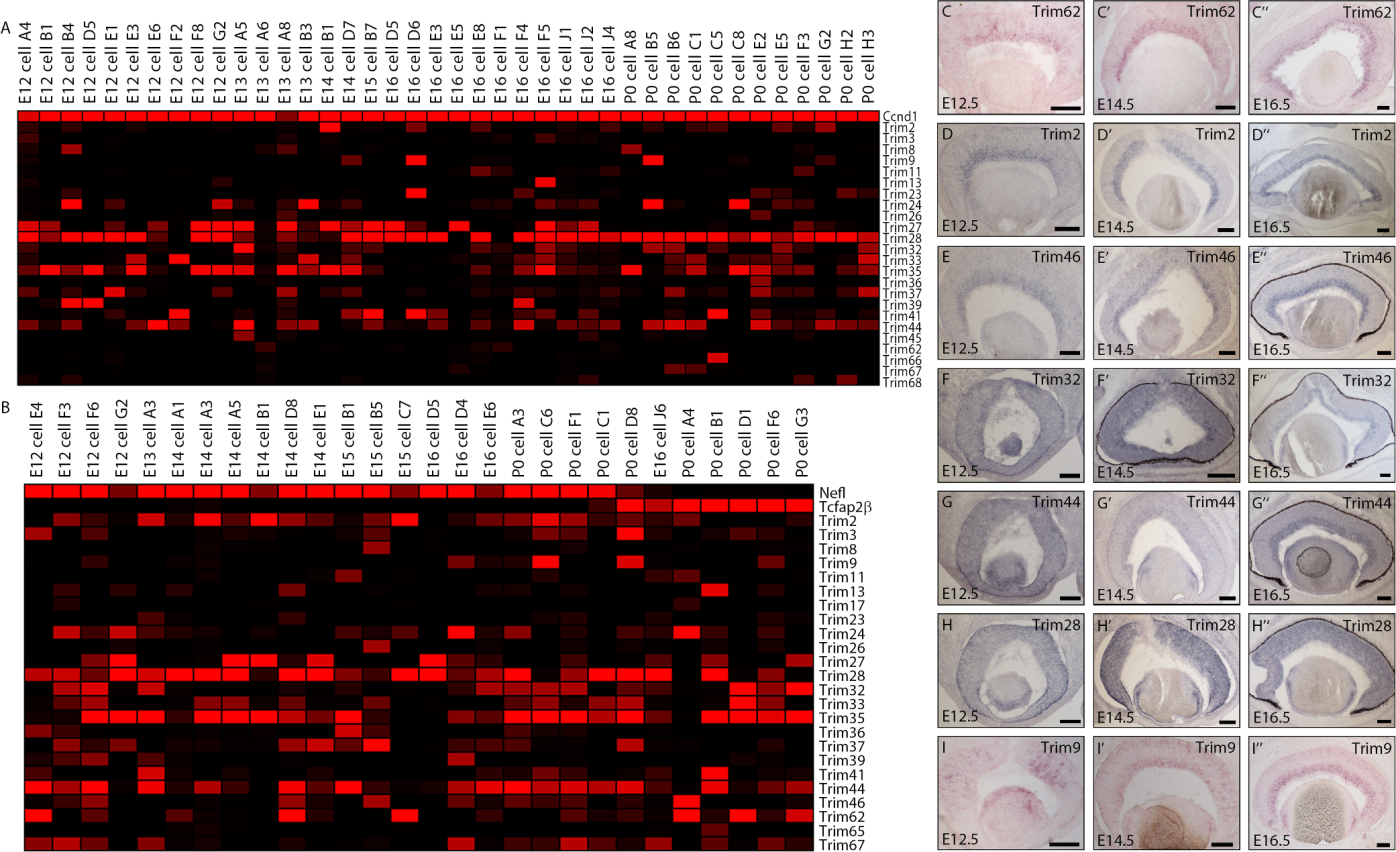
The TRIM family of genes is expressed in the developing mouse retina. A Genesis produced heatmap representing the microarray expression of the TRIM family of genes in (A) single cycling retinal progenitor cells (*Ccnd1*+), (B) developing retinal ganglion cells (*Nefl*+) and amacrine cells (*Tcfap2β*+) isolated from the developing mouse retina. The intensities of Affymetrix signals have been scaled such that a signal of 0 corresponds to a black color and a signal of 10000 corresponds to a bright red color. (C-I”) *In sit*u hybridization was performed using the following probes: *Trim62* (C-C”), *Trim2* (D-D”), *Trim46* (E-E”), *Trim32* (F-F”), *Trim44* (G-G”), *Trim28* (H-H”) and *Trim9* (I-I”) on retinal sections at E12.5, E14.5, E16.5. Scale bars represent 100 μm.

To gain insight into the mechanisms of cell fate determination operating within the retina, single cells were isolated from mouse retinas at various developmental timepoints and microarray hybridization was performed[10]. TRIM/RING-B-box-coiled-coil (RBCC) proteins are involved in a wide range of developmental processes and therefore implicated in several pathological conditions from genetic diseases to cancer development[36]. Deletion of *Trim2* in mice led to a reduction in the number of retinal ganglion cells later in life, but any phenotypes associated with other TRIM family genes have not been reported[37]. Therefore, we began by assessing the expression of *Trim* genes in the single cell transcriptomes. The first question we sought to address was which TRIM family genes were expressed in RPCs. As *CyclinD1* (*Ccnd1*) is a marker of cycling RPCs[38] we first examined the transcriptomes that expressed *Ccnd1* at various stages of development [E12.5, E13.5, E14.5, E15.5, E16.5, and post-natal day (P)0]. We found many members of the *Trim* family widely expressed in RPC transcriptomes (Fig 1A). More specifically, the family members *Trim28*, *Trim35 and Trim44* showed the most prominent expression among the highest number of RPCs (Fig 1A). Many of the *Trim* family genes displayed more heterogeneous expression patterns, appearing in everything from a majority of RPC profiles (e.g. *Trim27*, *Trim32*, and *Trim41*) to appearing in very few RPC profiles (e.g., *Trim3*, *Trim8*, *Trim11*, *Trim 62 and Trim66)*.

To further characterize the expression of the *Trim* genes in the developing retina, we next focused on their expression in subsets of single cells that had been classified as either developing amacrine or ganglion cells[30] (Fig 1B). For reference, we show *Neurofilament-light* (*Nefl*) as a marker of developing RGCs[39] and *Tcfap2β* as a marker of developing amacrines[29]. Of all the genes examined, only *Trim36 and Trim39* were found to be expressed only in *Nefl*+ cells and absent from *Tcfap2β*+ cells (Fig 1B) and even these two genes were only observed in a subset of the *Nefl*+ profiles. The majority of the *Trim* family genes observed in these cells were found in subsets of both *Nefl*+ and *Tcfap2β*+ cells, perhaps indicating a role for this family in general retinal neuronal development.

To further characterize the expression of the *Trim* genes, *in situ* hybridization (ISH) was performed on retinal cryosections derived from embryonic mice at three developmental stages (Fig 1C–1I”). Consistent with our single cell transcriptomic results, we observed a number of *Trim* family genes robustly expressed within developing retinal neurons (inner neuroblastic layer [INBL])[40] and the RPCs (outer neuroblastic layer [ONBL])[40] at the developmental stages, E12.5, E14.5 and E16.5. The expression of *Trim62* was found in subsets of cells in the INBL and was not detected in the ONBL (Fig 1C, 1C’ and 1C”). The genes *Trim2* and *Trim46* were expressed throughout development specifically in the INBL (Fig 1D, 1D’, 1D”, 1E, 1E’ and 1E”). *Trim32* was found to be weakly expressed in both the INBL and ONBL at E12.5 and E14.5, before finally localizing to the INBL at E16.5 (Fig 1F, 1F’ and 1F”). *Trim44* expression was also diffuse throughout the retina at E12.5 before localizing weakly to the INBL at E14.5 and eventually becoming more prominent in the INBL at E16.5 (Fig 1G, 1G’ and 1G”). *Trim28* expression differed from that of the other *Trim* genes investigated in this study in that it remained dispersed throughout both ONBL and INBL at all stages of development examined (Fig 1H, 1H’ and 1H”).

Since *Atoh7* has been shown to play an important role in the development of early born retinal neurons[12–16], *Atoh7* positive single cells were isolated from developing mouse retinas and their transcriptomes examined in an effort to identify new retinal cell fate regulators. We observed a significant number of *Trim* family genes expressed in these *Atoh7*+ single cell transcriptomes as well (S1 Fig), similar to our results in RPCs and in *Nefl*+/*Tcfap2β*+ cells. To identify potential new cell fate regulators, we utilized hierarchical clustering to reveal the genes most closely associated with *Atoh7* expression. In this analysis, we discovered that the most closely associated *Trim* family member was *Trim9*. Even though *Atoh7* and *Trim9* were significantly correlated, a closer examination of the single cell transcriptomes showed that *Trim9* was expressed only in a small subset of *Atoh7*+ cells, perhaps indicating either that *Trim9* expression is quite dynamic or that this gene only plays a functional role in a subset of retinal neurons. To examine the expression of Trim9 in more detail, ISH was again employed. At E12.5, *Trim9* was expressed in subsets of cells in the ONBL (Fig 1I). By E14.5, its expression was confined to the INBL and the outer portion of the ONBL (Fig 1I’), where mitoses are occurring and where newborn cone photoreceptors reside. By E16.5, *Trim9* expression was limited to the INBL (Fig 1I”). Given the expression pattern of *Trim9* and its association with *Atoh7*, we explored its potential role in early cell fate specification in the retina further.

### Characterization of *Trim9* KO retinas

**Fig 2:**
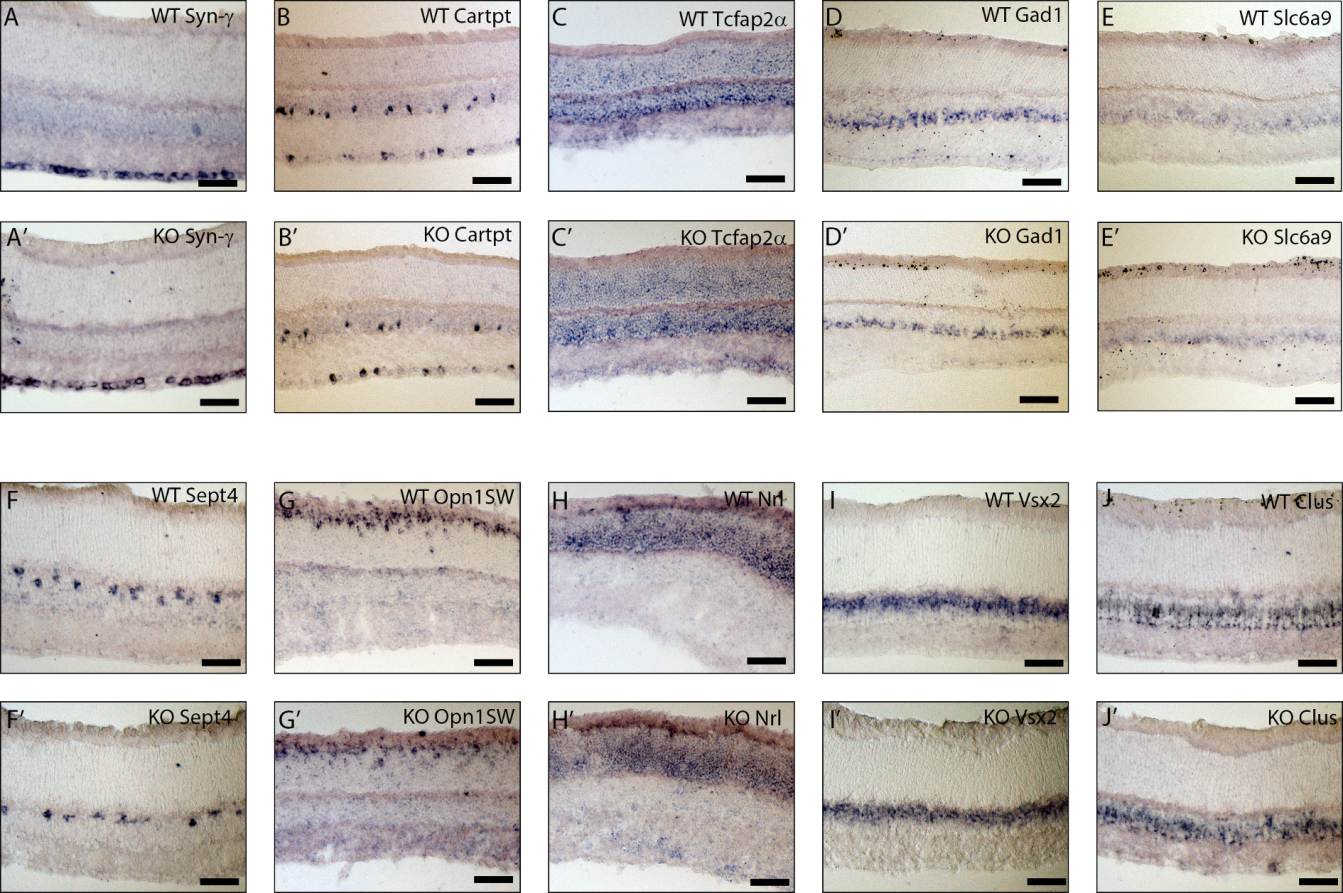
Characterization of retinal neurons in adult WT and *Trim9* deficient mouse retinas using *in situ* hybridization. *In situ* hybridization was used to identify populations of retinal neurons in WT and *Trim9* knockout littermates. Probes staining all RGCs (*Syn-γ;* A, A’), a subset of RGCs (*Cartpt;* B, B’), amacrine cells (*Tcfap2α;* C, C’), GABAergic amacrine cells (*Gad1;* D, D’), glycinergic amacrine cells (*Slc6a9;* E, E’), horizontal cells (*Sept4;* F, F’), short-wave cones (*Opn1SW;* G, G’), rod photoreceptors (*Nrl;* H, H’), bipolar cells (*Vsx2;* I, I’), and Müller glia (*Clus;* J, J’) were employed. Scale bars represent 100 μm.

Given the expression of *Trim9* in a subset of single developing *Atoh7*+ RPCs and its expression in the developing retina (Fig 1), we hypothesized that *Trim9* would play a role in cell fate determination within the retina. To address this possibility, we obtained a *Trim9* knockout mouse and examined the expression of markers of ganglion cells, amacrine cells, cones and horizontal cells in mature *Trim9* deficient retinas. We focused on the early-generated retinal neurons initially because of the expression of *Trim9* and because the phenotypes of *Atoh7* deficient animals are primarily associated with early born retinal neurons[12–16,20]. First, we used ISH on adult wildtype and *Trim9* knockout retinal cryosections to assess whether the absence of *Trim9* affected the development of early born retinal neurons (Fig 2). We examined populations of ganglion cells using probes to synuclein-γ *(Syn-*γ*)*[29,41] and *Cartpt*[42](Fig 2A, 2A’, 2B and 2B’), amacrine cells by expression of *Tcfap2α*[43](Fig 2C and 2C’), GABAergic amacrine cells by means of *Gad1*[44](Fig 2D and 2D’), glycinergic amacrine cells by *Slc6a9* (Fig 2E and 2E’)[44], horizontal cells with a probe to *Septin4 (Sept4)*[40](Fig 2F and 2F’), cone photoreceptors by opsin1, short wavelength sensitive (*Opn1SW*) (Fig 2G and 2G’), rod photoreceptors by expression of *Nrl*[45](Fig 2H and 2H’), bipolar cells by *Vsx2*[30](Fig 2I and 2I’), and Müller glia by *Clusterin (Clus)*[40](Fig 2J and 2J’). However, no major changes were observed in these primary classes and subclasses of retinal neurons in the *Trim9* deficient retinas.

**Fig 3:**
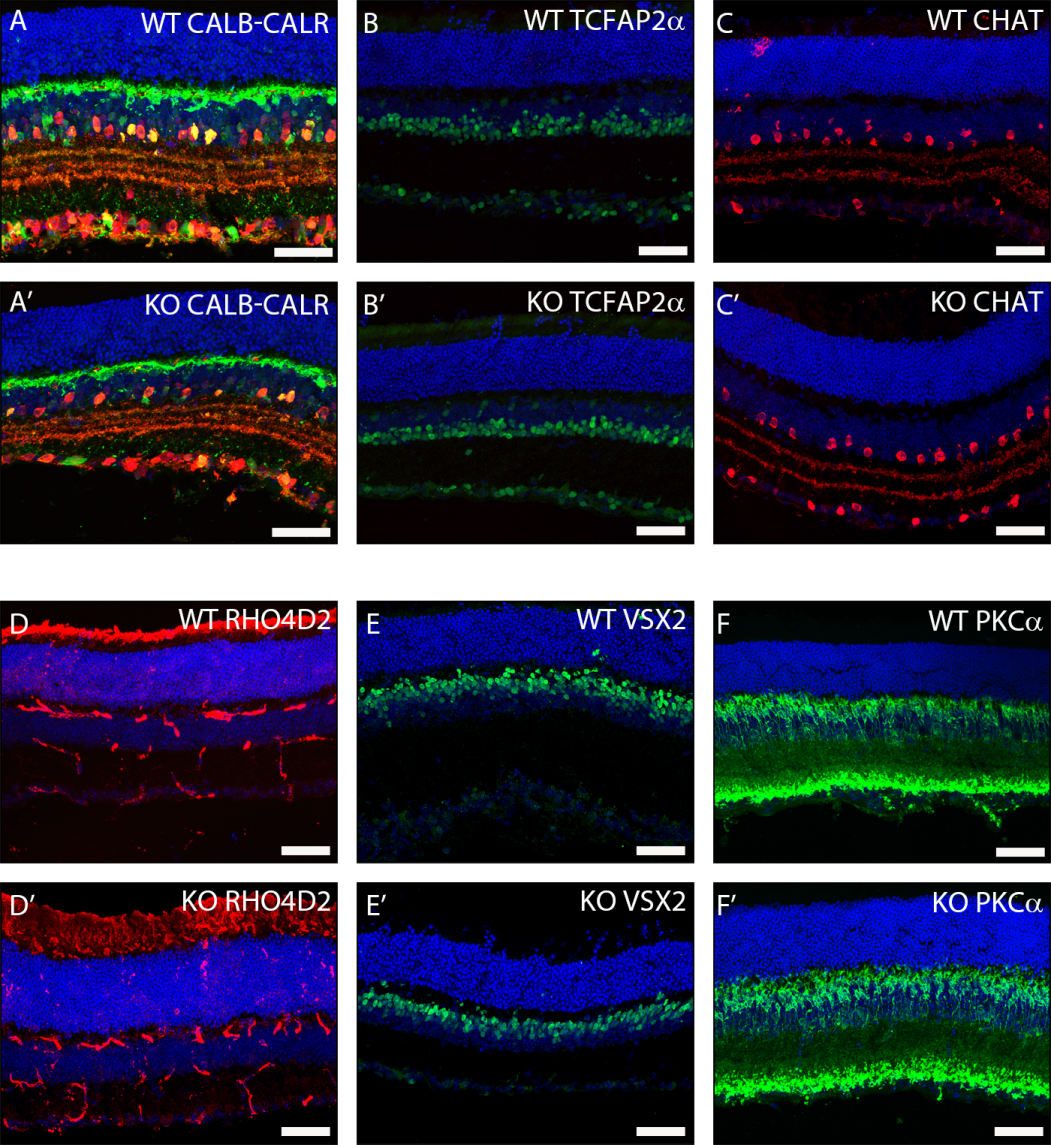
Morphological characterization of adult WT and *Trim9* deficient mouse retinas using immunohistochemistry on cryosections. Immunohistochemistry for different retinal neurons was performed on adult cryosections from WT and *Trim9* knockout littermates using antibodies for RGCs, amacrine cells and horizontal cells (anti-CALB and anti-CALR; A, A’), amacrine cells (anti-TCFAP2α; B, B’), cholinergic amacrine cells (anti-CHAT; C, C’), rod photoreceptors (anti- RHO4D2; D, D’), bipolar cells (anti-VSX2; E, E’ and anti-PKCα; F, F’). DAPI (blue) shows nuclear staining. Scale bars represent 50 μm.

Next, we wished to assess if there were any changes in broad populations of retinal neurons that we failed to detect by *in situ* hybridization. We thus performed antibody stains on retinal cryosections from *Trim9* deficient mice and their wildtype littermates. We inspected populations of horizontal, amacrine and ganglion cells (antibodies to CALB[44] and CALR[44], Fig 3A and 3A’), amacrine cells (antibodies to TCFAP2α[43] and CHAT)[46], Fig 3B, 3B’, 3C and 3C’), rod photoreceptors (anti-RHO4D2, Fig 3D and 3D’) and bipolar cells (antibodies to VSX2[30] and PKCα[44], Fig 3E, 3E’, 3F and 3F’). The *Trim9* knockout mice failed to display any significant changes in their populations of retinal neurons when compared to their wildtype littermates.

**Fig 4:**
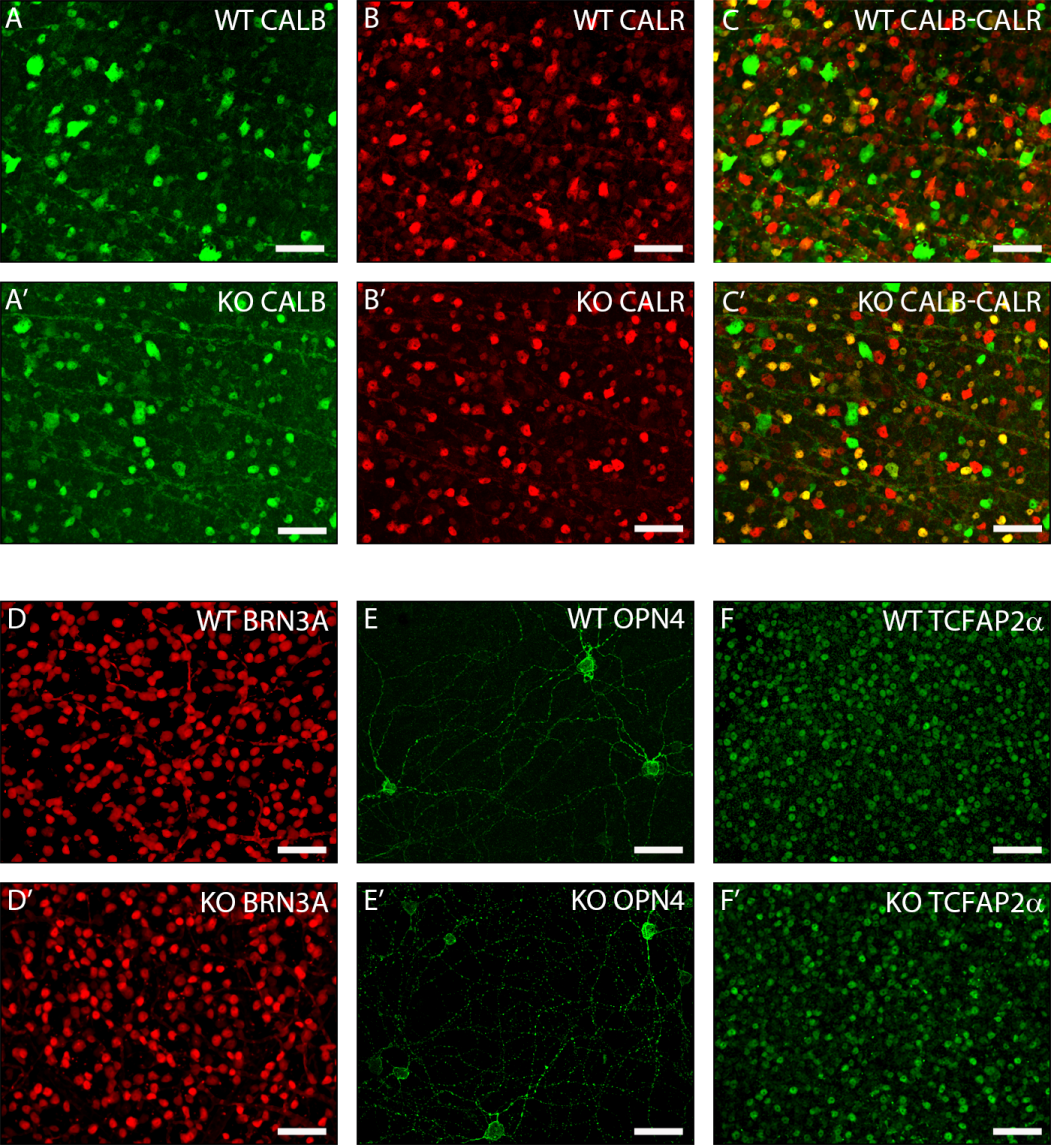
Quantitative assessment of retinal neurons in adult WT and *Trim9* deficient mouse retinas using immunohistochemistry on retinal flat-mounts. Immunohistochemistry was performed on flat-mounted adult retinas from WT and Trim9 knockout littermates. Confocal scans were performed on four different quadrants from each retina and the number of stained cells counted. Representative quadrant-matched images are shown. Antibodies for RGCs, amacrine cells and horizontal cells (anti-CALB and anti-CALR; A, A’- C, C’), a subset of RGCs (anti-BRN3A; D, D’), intrinsically sensitive RGCs (anti-OPN4; E, E’) and amacrine cells (anti-TCFAP2α; F, F’). DAPI (blue) shows nuclear staining. Scale bars represent 50 μm.

It is possible that any smaller differences might have been difficult to detect on an initial screen of retinal sections. Therefore, to more rigorously quantify any differences in retinal cell number between the wildtype and *Trim9* knockout mice, we performed immunohistochemistry on flat-mounted retinas. For each antibody used, we separated the retina into four different quadrants and quantified using images taken from each distinct quadrant. We used anti-CALB and anti-CALR antibodies to visualize a combination of amacrine, horizontal and ganglion cells, since these comprise most of the major early born cell types of the retina (Fig 4A–4C’). *Brn3a* has been shown to be expressed by a large fraction of RGCs[47], while OPN4 labels a subset of melanopsin positive ganglion cells[48]. *Tcfap2α* is a characterized marker of amacrine cells[43]. Thus, we used anti-BRN3A, anti-OPN4 and anti-TCFAP2α antibodies to visualize populations of RGCs and amacrine cells (Fig 4D–4F’). Counting the cell numbers and comparing three separate retinas from wildtype and *Trim9* deficient littermates revealed no significant differences between the numbers of different cell types (Table 1).

**Table 1:** Quantification of neuronal populations using flat-mount immunohistochemistry.

Antibody	Average # of cells in WT ± SEM	Average # of cells in KO ± SEM	P-value
Anti-CALR	196.66 ± 12.77	148 ± 14.14	0.38793459
Anti-CALB	193.33 ± 38.42	265.33 ± 29.24	0.210249502
Anti-BRN3A	338.25 ± 40.55	344.25 ± 41.90	0.921395545
Anti-TCFAP2α	367.25 ± 97.48	356.25 ± 90.14	0.936669398
Anti-OPN4	9.75 ± 1.65s	9.5 ± 1.7	0.977444685

Immunohistochemistry was performed on flat-mounted adult retinas from WT and *Trim9* knockout littermates using antibodies to CALR, CALB, BRN3A, TCFAP2α and OPN4. Retinal neurons were counted manually from four fields of view. Average cell counts with standard errors of mean (SEM) and P-value are shown in Table 1.

Since *Trim9* is expressed during development, it is conceivable that a phenotype may manifest itself early and then be compensated for later. Therefore, we examined developing wildtype and *Trim9* knockout retinas at embryonic stages. *In situ* hybridization was performed on cryosections of embryonic day (E)14.5 pups. Probes specific for developing photoreceptors (*Otx2*[49]), developing ganglion cells (*Syn-γ* and *Ebf3*[50]), and RPCs (*Vsx2*[30]) were examined. Despite the robust expression of *Trim9* at this stage, no major changes were observed in each of these developing neuronal populations (data not shown).

**Fig 5:**
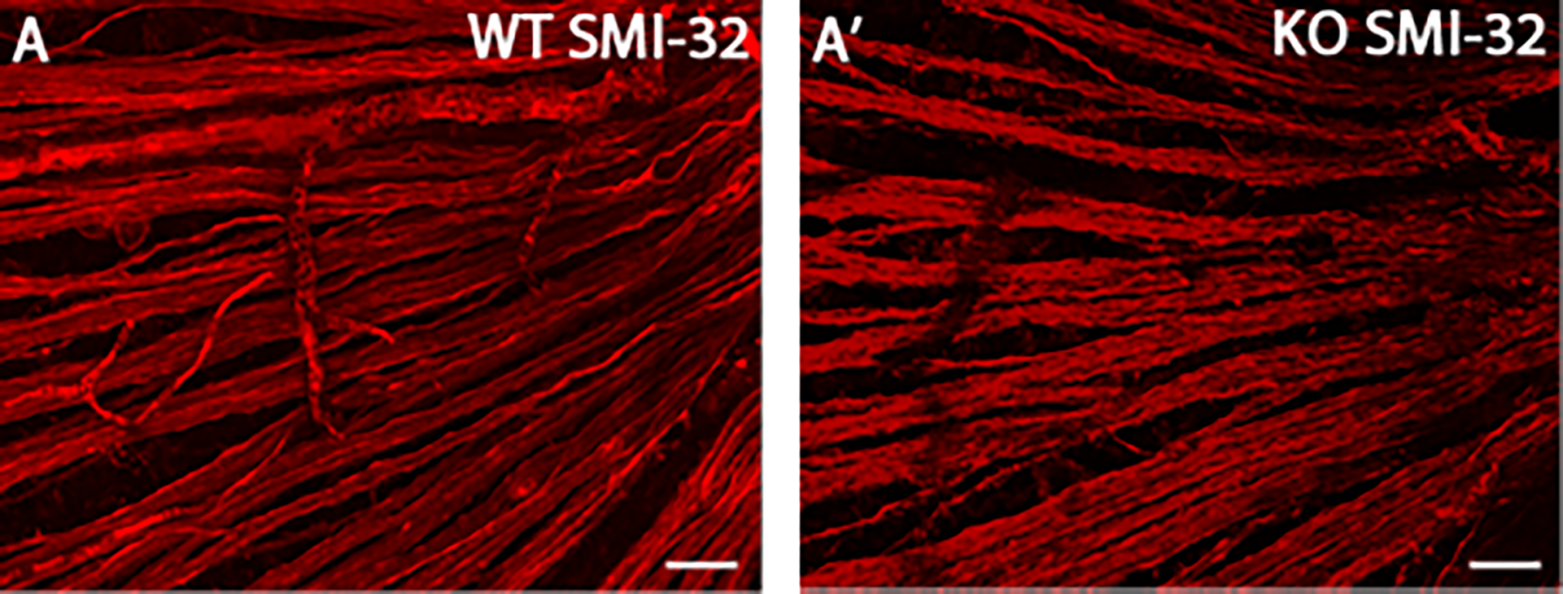
Visualization of RGCs and their processes in adult WT and *Trim9* deficient flat-mounted mouse retinas. Retinas obtained from WT and *Trim9* knockout mice were flat-mounted and immunohistochemistry was performed using the anti-SMI-32 antibody (A, A’). Scale bars represent 50 μm.

Since our observations failed to support our hypothesis that *Trim9* plays a role in cell fate determination in the retina, we decided to examine other aspects of retinal development such as RGC morphology. As it was previously reported that *Trim9* plays a role in axon branching and guidance in the cerebral cortex[26], we hypothesized that the retinas of *Trim9* deficient mice may show defects in axon morphology or neurite branching. In order to better visualize a large subset of retinal ganglion cells, we used an antibody to SMI-32, a heavy chain non-phosphorylated neurofilament protein component of axons that labels alpha-RGCs[51,52]. Again, we failed to observe any reproducible changes in the morphologies of alpha-RGCs (Fig 5A and 5A’).

**Fig 6:**
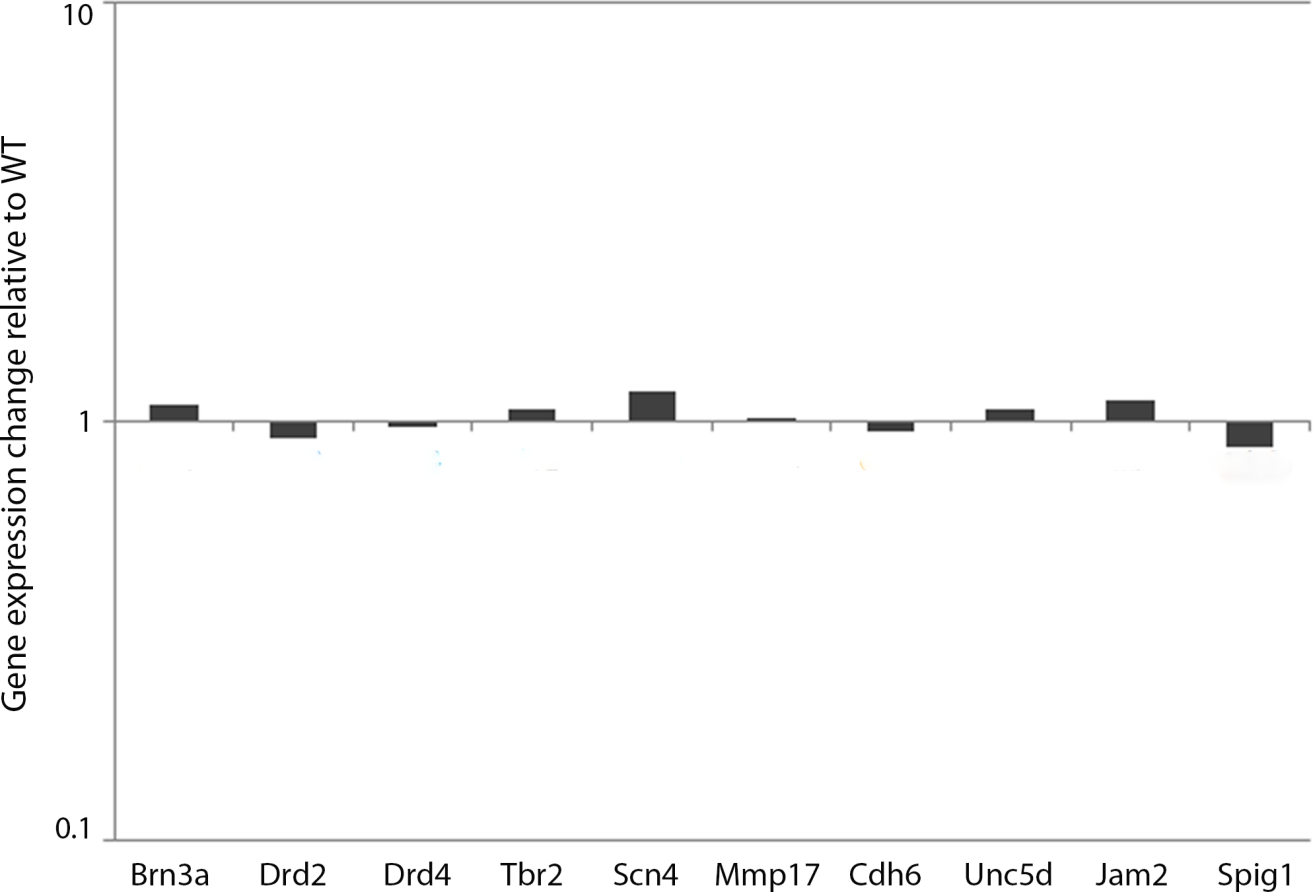
Examination of RGC subset genes in WT and *Trim9* deficient mice using quantitative real time PCR (qPCR). RNA was isolated from retinas of adult WT and *Trim9* knockout mice and qPCR was performed using primers specific for RGC subsets marked by expression of the genes *Brn3a*, *Drd2*, *Drd4*, *Tbr2*, *Scn4*, *Mmp17*, *Cdh6*, *Unc5d*, *Jam2* and *Spig1*. Error bars indicating standard deviation were calculated in Microsoft Excel.

The single cell transcriptome data showing that *Trim9* is only found in a subset of *Atoh7*+ cells (S1 Fig) raised the possibility that subsets of developing ganglion cells may be affected in the retinas of *Trim9* deficient mice, rather than the whole population of RGCs. Since RGCs are a small percentage of retinal cells, a phenotype confined to a specific subtype may have escaped detection when examining whole retinas by *in situ* hybridization or immunohistochemistry. Thus, we employed quantitative real time PCR (qPCR) to characterize potential differences in subsets of ganglion cells, utilizing specific markers of ganglion cell subsets. These included *Brn3a*, *Drd2*, *Drd4*, *Tbr2*, *Scn4*, *Mmp17*, *Cdh6*, *Unc5d*, *Jam2*, and *Spig1*. *Brn3a*, a gene encoding a POU-domain containing transcription factor is a marker of ganglion cells that project to the contralateral superior colliculus and the dorsal lateral geniculate nucleus[53]. *Drd4* expression marks the ON-OFF direction sensitive ganglion cells (DSGCs) that prefer nasal motion[54]. *Tbr2* is a selective marker of the intrinsically photosensitive ganglion cell population expressing *Opn4*[55]. *Mmp17* and *Cdh6* are reported markers of the ON-OFF DSGCs[42]. A gene encoding a netrin receptor, *Unc5d* is expressed by ON RGCs[55]. *Jam2* labels a population of OFF RGCs, also called J-RGCs[56]. *Spig1* is a known marker of ON DSGCs[42]. The expression of all these genes remained unchanged in the *Trim9* KO retina when compared to wild type littermates (Fig 6), indicating that loss of *Trim9* does not affect the identifiable subsets of RGCs in the retina.

Finally, since we did not detect any developmental phenotypes in *Trim9* deficient retinas, we decided to employ a more unbiased approach and performed a transcriptomic analysis of *Trim9* mutant retinas compared to WT retinas. Our belief was that any changes at the transcriptional level could lead to the discovery of unpredicted phenotypes in the *Trim9* deficient retinas. To that end, we performed microarrays on adult WT and *Trim9* knockout littermates (n = 3) (full data set available from the Gene Expression Omnibus, GEO database NCBI, accession number: GSE114323). The downregulated genes on this list did not point to a discrete cell fate phenotype and were potentially involved in a diverse number of processes. To more thoroughly examine the microarray data, gene ontology analysis was performed on the 100 most downregulated and upregulated genes (with approximately a 1.5 to two-fold change in expression levels) in the *Trim9* knockout retina and genes were classified according to biological process (Table 2), cellular component (Table 3) and molecular function (Table 4). Among the biological processes affected were catabolic processes (enrichment FDR = 0.001–0.003) and apoptotic and cell death pathways (enrichment FDR = 0.0037–0.0098). Cellular component analysis indicated that the gene products were located predominantly in the nucleolus (enrichment FDR = 0.0009) or were part of proteasome (enrichment FDR = 0.0005), catalytic (enrichment FDR = 0.0005) or endopeptidase complexes (enrichment FDR = 0.0005). Functionally, gene products were involved in RNA or protein binding (enrichment FDR = 0.0003) or ubiquitin protein ligase binding (enrichment FDR = 0.0197). While these downregulated genes provide some future avenues to explore, they did not definitively point to a specific phenotype for *Trim9* in the retina. In contrast to the downregulated genes, the genes upregulated in *Trim9* KO retinas were not observed as significantly involved in any particular process.

**Table 2:** Gene ontology analysis.

Enrichment FDR	Genes in list	Total genes	Functional Category
0.0010	18	1285	Cellular catabolic process
0.0010	14	785	Cellular macromolecule catabolic process
0.0021	19	1541	Organic substance catabolic process
0.0027	19	1602	Catabolic process
0.0037	14	963	Macromolecule catabolic process
0.0037	11	620	Cellular protein catabolic process
0.0037	7	229	Neuron apoptotic process
0.0037	8	312	Neuron death
0.0054	4	56	Cellular aldehyde metabolic process
0.0054	18	1660	Apoptotic process
0.0054	10	555	Apoptotic signaling pathway
0.0054	7	259	Intrinsic apoptotic signaling pathway
0.0058	18	1685	Programmed cell death
0.0066	6	190	Regulation of cellular protein catabolic process
0.0084	2	5	Methylglyoxal metabolic process
0.0084	11	740	Protein catabolic process
0.0098	18	1793	Cell death
0.0104	7	308	Regulation of cellular catabolic process
0.0115	16	1517	Cellular response to stress
0.0115	5	144	Negative regulation of neuron apoptotic process
0.0120	6	229	Steroid metabolic process
0.0123	2	7	Regulation of miRNA metabolic process
0.0192	12	992	Negative regulation of molecular function
0.0192	2	9	Aldehyde catabolic process
0.0193	3	41	Intrinsic apoptotic signaling pathway in response to oxidative stress
0.0220	3	44	RNA secondary structure unwinding
0.0220	14	1329	Regulation of apoptotic process
0.0228	6	275	Regulation of neuron death
0.0228	14	1343	Regulation of programmed cell death
0.0255	5	189	Negative regulation of neuron death

Microarray results were analyzed using the Bioconductor Affy package in R. RMA was employed for background adjustment and normalization[33]. The top hundred genes downregulated in the *Trim9* KO retinas obtained by microarray analysis were further classified using the ShinyGO v0.41 Gene Ontology Enrichment Analysis tool according to biological process. Enrichment FDR denotes the false discovery rate.

**Table 3:** Gene ontology analysis.

Enrichment FDR	Genes in list	Total genes	Functional Category
0.0009	14	871	Nucleolus
0.0050	4	71	Proteasome complex
0.0050	14	1121	Catalytic complex
0.0050	4	71	Endopeptidase complex
0.0113	4	93	Peptidase complex
0.0206	5	197	Myelin sheath
0.0491	6	361	Microtubule

Microarray results were analyzed using the Bioconductor Affy package in R. RMA was employed for background adjustment and normalization[33]. The top hundred genes downregulated in the *Trim9* KO retinas obtained by microarray analysis were further classified using the ShinyGO v0.41 Gene Ontology Enrichment Analysis tool according to cellular component.

**Table 4:** Gene ontology analysis.

Enrichment FDR	Genes in list	Total genes	Functional Category
0.0003	20	1582	RNA binding
0.0003	18	1353	Identical protein binding
0.0024	19	1771	Enzyme binding
0.0042	2	5	Ribonuclease III activity
0.0042	2	5	Double-stranded RNA-specific ribonuclease activity
0.0098	2	8	Intramolecular oxidoreductase activity interconverting aldoses and ketoses
0.0197	6	291	Ubiquitin protein ligase binding
0.0197	6	296	Ubiquitin-like protein ligase binding
0.0240	3	63	RNA helicase activity
0.0240	3	63	ATP-dependent RNA helicase activity
0.0240	3	64	RNA-dependent ATPase activity
0.0240	9	702	Protein homodimerization activity
0.0261	8	595	Protein domain specific binding
0.0336	3	78	Cysteine-type endopeptidase activity
0.0336	4	156	Phosphatase binding
0.0375	2	25	Intramolecular transferase activity
0.0381	3	87	Ribonuclease activity
0.0381	2	26	Endoribonuclease activity producing 5 -phosphomonoesters
0.0382	4	179	Protein C-terminus binding
0.0382	3	95	ATP-dependent helicase activity
0.0382	4	180	Cysteine-type peptidase activity
0.0382	11	1171	Protein dimerization activity
0.0382	2	31	ATPase regulator activity
0.0382	3	95	Purine NTP-dependent helicase activity
0.0429	2	35	Peptidase activator activity
0.0429	2	35	Endonuclease activity active with either ribo- or deoxyribonucleic acids and producing 5 -phosphomonoesters
0.0429	3	108	Ubiquitin-like protein-specific protease activity
0.0429	7	587	Kinase binding
0.0429	9	894	Enzyme regulator activity
0.0480	3	114	Protein phosphatase binding

Microarray results were analyzed using the Bioconductor Affy package in R. RMA was employed for background adjustment and normalization[33]. The top hundred genes downregulated in the *Trim9* KO retinas obtained by microarray analysis were further classified using the ShinyGO v0.41 Gene Ontology Enrichment Analysis tool according to molecular function.

## Discussion

To gain a better understanding of retinal progenitor cell behavior, we examined the expression of 33 genes belonging to the TRIM family in single cell transcriptomes generated from the developing mouse retina[10,29]. We found robust expression of a significant number of *Trim* genes in RPCs and developing neurons between E12.5 and E16.5 and corroborated these results by ISH. Of the 33 *Trim* genes queried, only *Trim9*, *Trim36 and Trim39* were found to be expressed in developing RGCs (*Nefl*+ cells) and completely absent from amacrine cells (*Tcfap2β*+ cells), and therefore potential candidates to specifically regulate RGC development. Furthermore, using hierarchical clustering, we found a significant correlation between the expression of *Atoh7* and *Trim9* in the single cell data. This correlation led us to hypothesize that *Trim9* would be involved in the development of retinal neurons, perhaps playing a crucial role in those neurons derived from an *Atoh7* lineage.

TRIM9, a member of the TRIM family of ubiquitin ligases, has been shown to play an important role in axon guidance and axonal branching in the mouse cerebral cortex[26–28]. After deletion of the *Trim9* gene, axons in the cerebral cortex and corpus callosum exhibit exaggerated axon branching[27]. Despite several studies investigating its function in the brain, the role of *Trim9* in the retina had not been characterized. To that end, we obtained *Trim9* deficient mice and used a combination of morphological and gene expression analyses to assess the role of *Trim9* during retinal development. Surprisingly, the production of early-born retinal neurons was not affected in *Trim9* knockout animals. More quantitative analyses for different subtypes of retinal ganglion cells also failed to show any differences in smaller subsets of distinct RGCs, similar to the larger scale studies. This observed lack of differences between WT and Trim9 deficient retinas was consistent across both embryonic and adult stages.

We hypothesize that the lack of phenotype in *Trim9* deficient retinas could be due to redundancy through the expression of other members of the TRIM family. Studies have shown that the loss of a gene in vertebrate systems is often compensated for by the function of related genes or family members[57]. For example, it was observed that the deletion of *Sox12* in mice, a member of the *SoxC* family that is highly expressed during embryogenesis, particularly within the peripheral and central nervous system, does not cause gross morphological changes or loss of fertility or viability[58]. Upon further investigation, it was observed that two other members belonging to the *SoxC* family, *Sox4* and *Sox11* work synergistically with *Sox12*[58]. The authors concluded that one reason for the lack of a phenotype could be functional compensation by SOX4 and SOX12[58]. An increase of expression of these two genes in some organs was observed, which supported this theory[58]. There are numerous other examples of this phenomenon as well. These include: knockouts of *Casp12* from the caspase gene family[58], *Adcy4* of the adenylate cyclase gene family[58], *Capn5* of the calpain gene family[59], and *Pmm1* of the phosphomannomutase gene family[60]. Similarly, in zebrafish, it has been shown that receptors of bone morphogenetic proteins (BMP), SMAD1 and SMAD9 function redundantly to mediate dorso-ventral patterning[61]. Although knockdown of one or the other does not lead to visible dorsalization, a double knockdown causes a strong phenotype[61]. Finally, even within our own lab, removing the kinase *Plk3* from the developing retina led to no discernible phenotype[23].

It has been proposed that approximately 15% of KO mice will not have a discernible phenotype[57]. Given this statistic and the fact that *Trim9* belongs to a very large gene family of approximately 40 genes[36], our lack of a phenotype is perhaps less surprising. Furthermore, as we demonstrate in this study, there is wide expression of the TRIM family of genes in the same single cells during early retinal development. This observation includes those single cells that express *Trim9*. Taken together, these results support our contention that some form of compensation may be at work. One additional possibility is that the loss of *Trim9* could trigger upregulation of other *Trim* genes, whereby these genes could now compensate for the loss of *Trim9*. However, our transcriptomic analysis of *Trim9* KO retinas did not show an upregulation of other TRIM family genes, with the caveat that this analysis was not performed across multiple developmental stages or at the single cell level.

Taken together our results demonstrate that solely inactivating *Trim9*, a gene whose expression was significantly correlated with *Atoh7* expression, does not lead to significant alterations in cell fate during retinal development. Furthermore, since Trim9 has been shown to be important for axon branching in the cerebral cortex[26,27], it is possible that the absence of *Trim9* leads to defects in the wiring and/or connections of retinal ganglion cells and their targets in the brain. Additionally, these results do not rule out the possibility that *Trim9* works in concert with other genes, either within or outside of its gene family, that may compensate for its loss of its function. Investigation into the remaining members of the *Trim* family may reveal roles for them in retinal development and function, possibly in concert with *Trim9*. However, with such a large number of TRIM family genes, these experiments are quite onerous. The discovery of genome editing, though, makes the challenge somewhat easier to overcome. Targeting several members of the *Trim* family of genes at the same time is possible using a multiplexed Clustered Regularly Interspaced Short Palindromic Repeats (CRISPR) genome editing-based strategy[62]. Successfully mutating different combinations of TRIM family genes in the presence of *Trim9* mutations would be a method to address these issues of possible compensation. In the end, some strategy such as this will be necessary to dissect the precise role of *Trim9* in the retina.

## Supporting information

S1 Fig The Trim family of genes is expressed in *Atoh7*+ single RPCs. A Genesis generated heatmap representing the microarray expression of the TRIM family of genes in single *Atoh7*+ cells isolated from the developing mouse retina at E12.5, E14.5 and E16.5. The intensities of Affymetrix signals have been scaled such that a signal of 0 corresponds to a black signal and a signal of 2500 corresponds to a bright red color.
(TIF)

S1 Table List of probes used for ISH. The sequences of the primers used to amplify specific genes from mouse retinal cDNA for generating RNA probes used for section ISH are shown in this table.
(XLSX)

S2 Table List of qPCR primers. The sequences of the primers used for qPCR are shown in this table.
(XLSX)
