# Supplementary figures and images for: The Trim family of genes and the retina: Expression and functional characterization
Source: PLoS One. 2018 Sep 12;13(9):e0202867. doi: 10.1371/journal.pone.0202867 (PMC6135365; doi:10.1371/journal.pone.0202867)

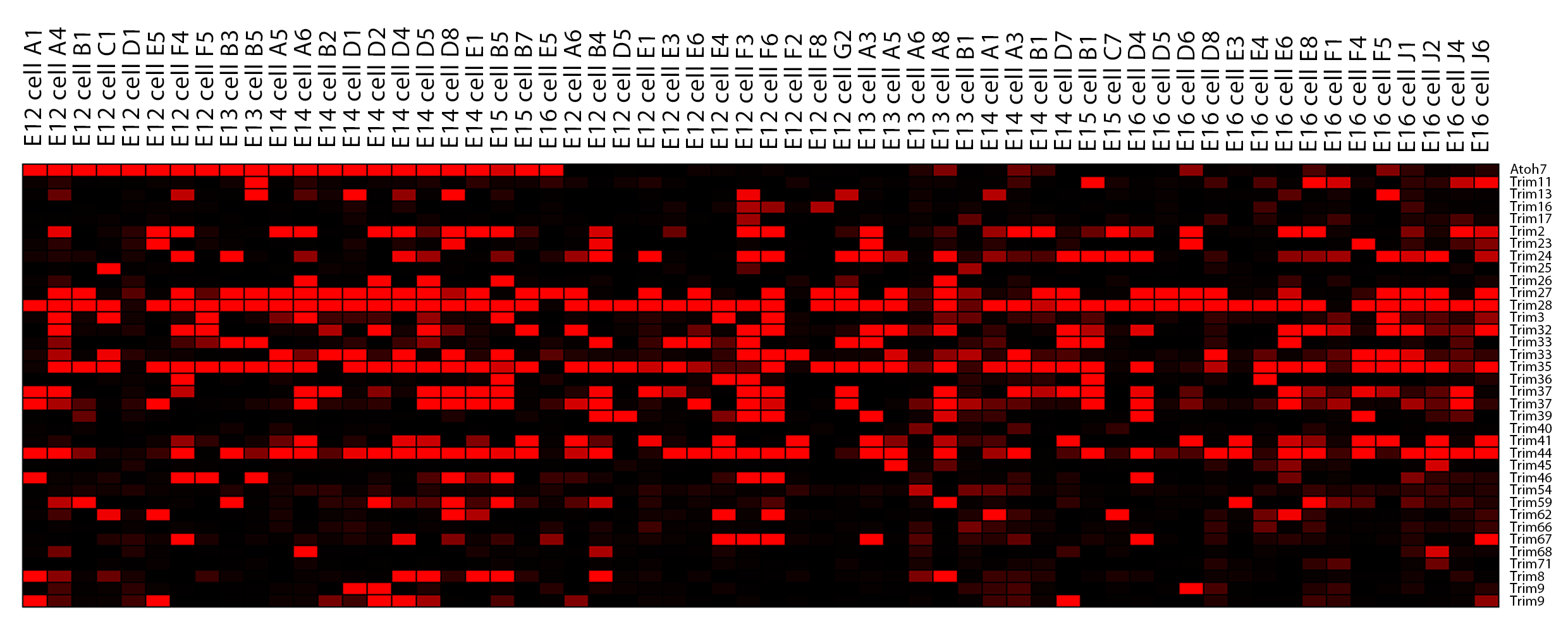

Supplement: S1 Fig — A Genesis generated heatmap representing the microarray expression of the TRIM family of genes in single Atoh7+ cells isolated from the developing mouse retina at E12.5, E14.5 and E16.5. The intensities of Affymetrix signals have been scaled such that a signal of 0 corresponds to a black signal and a signal of 2500 corresponds to a bright red color. (TIF) [file pone.0202867.s001.tif]
